# Repetitive Transcranial Magnetic Stimulation at Different Sites for Dysphagia After Stroke: A Randomized, Observer-Blind Clinical Trial

**DOI:** 10.3389/fneur.2021.625683

**Published:** 2021-05-26

**Authors:** Lida Zhong, Jinzhu Rao, Jing Wang, Fang Li, Yang Peng, Huiyu Liu, Yan Zhang, Pu Wang

**Affiliations:** ^1^Department of Rehabilitation Medicine, Yue Bei People's Hospital, Shaoguan, China; ^2^School of Educational Science, Huazhong University of Science and Technology, Wuhan, China; ^3^Department of Rehabilitation Medicine, The Seventh Affiliated Hospital Sun Yat-sen University, Shenzhen, China

**Keywords:** repetitive transcranial magnetic stimulation, dysphagia, stroke, cerebellum, mylohyoid cortical

## Abstract

**Background:** The clinical efficacy of repetitive transcranial magnetic stimulation (rTMS) protocols on patients with poststroke dysphagia is still unclear.

**Objective:** This trial aimed to explore and analyze the effectiveness of 5 Hz rTMS on the unaffected hemisphere, affected hemisphere, and cerebellum in stroke patients with dysphagia.

**Methods:** This observer-blind and randomized controlled trial included a total of 147 patients with stroke. Patients were divided into four treatment groups: the unaffected hemispheric group, the affected hemispheric group, the cerebellum group and the control group. Each group received traditional dysphagia treatment 5 days a week for 2 weeks. All recruited patients except for those in the control group underwent 10 consecutive rTMS sessions for 2 weeks. For the affected hemispheric group and unaffected hemispheric group, 5 Hz rTMS was applied to the affected mylohyoid cortical region or to the unaffected mylohyoid cortical region. For the cerebellum group, 5 Hz rTMS was applied to the mylohyoid cortical representation of the cerebellum (4.3 cm lateral and 2.4 cm below the inion). The Fiberoptic Endoscopic Dysphagia Severity Scale (FEDSS), Penetration/Aspiration Scale (PAS), Gugging Swallowing Screen (GUSS), and Standardized Swallowing Assessment (SSA) were used to evaluate clinical swallowing function before the intervention (baseline), immediately after the intervention and 2 weeks after the intervention.

**Results:** There were significant time and intervention interaction effects on the FEDSS, PAS, SSA, and GUSS scores (*p* < 0.05). In a direct comparison of the swallowing parameters of the four groups, the changes in FEDSS, PAS, SSA, and GUSS scores showed a significantly greater improvement in the unaffected hemispheric group, the affected hemispheric group and cerebellum group than in the control group (*p* < 0.05).

**Conclusions:** Whether stimulating the unaffected hemisphere or the affected hemisphere, 5 Hz high-frequency rTMS on mylohyoid cortical tissue might have a positive effect on poststroke patients with dysphagia. In addition, cerebellar rTMS is a safe method that represents a potential treatment for poststroke dysphagia, and more clinical trials are needed to develop this technique further.

**Clinical Trial Registration:** chictr.org.cn, identifier: ChiCTR2000032255.

## Introduction

Dysphagia, affecting 27–64% of stroke patients, is one of the most common poststroke sequelae (1) and is often associated with malnutrition, pneumonia, and dehydration (2). Conventional therapies for dysphagia include postural interventions, swallowing maneuvers, and exercises. Even though the above treatments have been widely applied in clinical practice, there is not enough clinical evidence to prove their efficacy (3–5). Recently, non-invasive cortical stimulation, a new strategy, has been used as a way of promoting neurologic rehabilitation after stroke. For example, transcranial magnetic stimulation is considered a well-tolerated technique that can modulate cortical excitability (6, 7). Moreover, repetitive transcranial magnetic stimulation (rTMS) of the motor cortex area related to swallowing directly induces the excitability of swallowing muscles regulated by corticobulbar projections (8), thereby enhancing swallowing function (9, 10). In patients with dysphagia after stroke, the application of 3 Hz (11) and 10 Hz (12) rTMS on the ipsilateral motor cortex represented by the esophageal or mylohyoid cortex showed significant improvement compared with sham stimulation. Meanwhile, both 1 Hz (13) and 5 Hz (9) rTMS on the contralateral motor cortex represented by the pharyngeal or mylohyoid cortex showed improved swallowing function. According to reports, rTMS showed different efficacies when patients with dysphagia were subjected to different stimulation parameters, such as intensity, frequency, and stimulation position.

It is controversial to stimulate either the ipsilesional or contralesional hemisphere. Previous systematic studies have shown different outcomes regarding the efficacy of non-invasive brain stimulation (NIBS) according to its stimulating point. Specifically, a review reported that no differences were found dependent on the stimulation site (14), whereas another study discovered that contralesional stimulation is better than ipsilesional stimulation (15). The latter study applied a combination of 5 Hz rTMS with pharyngeal electrical stimulation on the contralesional hemisphere (16). In conclusion, previous reviews reported different results because of the various stimulation applications, and it was relatively difficult to confirm whether the effect of contralesional rTMS was better than ipsilesional rTMS in regard to improving swallowing function.

Cerebellar neurostimulation has been considered an unexplored method and a prelude of treatment for dysphagia by modulating swallowing pathways. It has been shown that the cerebellum can be strongly activated during swallowing exercise (17), and stimulation of the cerebellum in the hemispheres or midline can induce different pharyngeal electromyography responses. For example, Sasegbon et al. (18) demonstrated that rTMS on the cerebellar vermis had inhibitory effects on pharyngeal motor cortical activity and swallowing behavior. Vasant et al. (19) demonstrated that hemispheric cerebellar rTMS increases cortical pharyngeal motor evoked potential (PMEP) amplitudes. Using the advantages of neuronavigation and comparing the latency and amplitude of pharyngeal motor evoked potentials, the authors confirmed the best position to obtain these responses, which was 4.3 cm lateral and 2.4 cm below the inion (19). Recently, some studies (20, 21) have explored the possibility of rTMS on cerebellar tissue in the treatment of dysphagia.

Therefore, this prospective, randomized, observer-blind clinical study focused on the effectiveness and safety of rTMS in stroke patients with dysphagia. Outcomes after stimulation of the unaffected side, the affected side and the cerebellum were compared to determine which area of stimulation is more beneficial for the recovery of patients with dysphagia to guide clinical work in the future.

## Materials and Methods

### Subjects

One hundred fifty-five poststroke patients suffering from dysphagia were included from April 2020 to April 2021. All of the patients were hospitalized to the Department of Rehabilitation Medicine, Yue Bei People's Hospital, Guangdong Province, China. The inclusion criteria were as follows: (1) subacute stroke <3 months diagnosed by imaging tests, including computed tomography (CT) or magnetic resonance imaging (MRI), hemorrhagic stroke or unilateral ischemia; (2) dysphagia confirmed by fiberoptic endoscopic evaluation of swallowing (FEES); and (3) no prior dysphagia rehabilitation. The exclusion criteria included history of any other neurogenic disease, epilepsy, tumor; severe cognitive impairment or aphasia; and contraindication to electrical or magnetic stimulation. All patients provided written informed consent before inclusion. The trial protocol was approved by the Ethics Committee of Yue Bei People's Hospital, and this clinical study was carried out and reported according to the Consolidated Standards of Reporting Trials (CONSORT) guidelines (22). Details of trial protocol registration can be seen in chictr.org.cn (chictr.org.cn Identifier: ChiCTR2000032255).

A total of 155 poststroke patients with dysphagia were recruited before assessment for eligibility, and 147 were included after exclusion.

**Figure 1:**
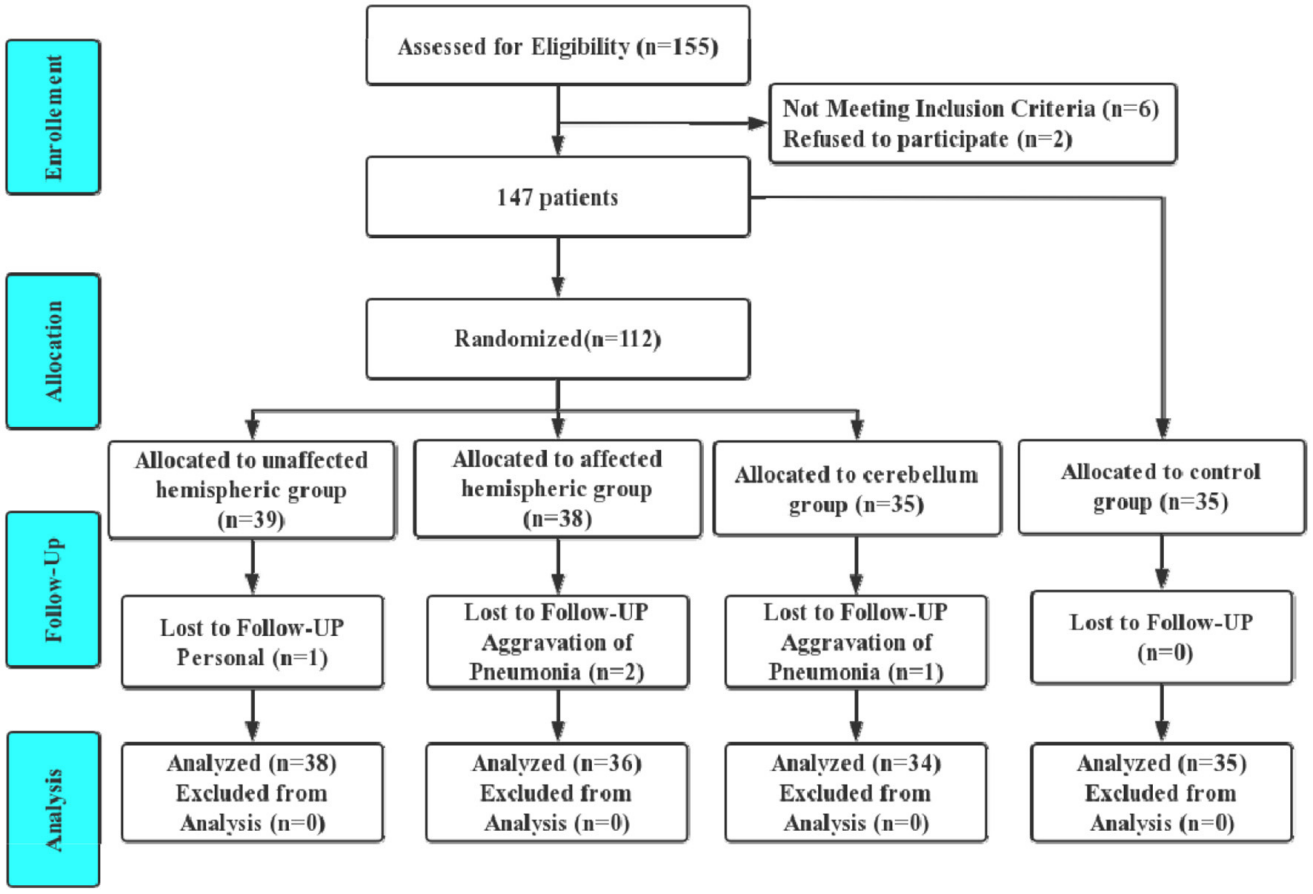
Participant flow diagram.

One hundred forty-seven patients were divided into four groups: the unaffected hemispheric group, affected hemispheric group, cerebellum group and control group. Four included patients withdrew from the trial. One patient in the unaffected hemispheric group withdrew for a personal reason not relevant to the trial. Two patients in the affected hemispheric group and one in the cerebellum group quit the study due to exacerbated pneumonia. Consequently, 143 patients completed the trial (Figure 1).

### Experimental Design

**Figure 2:**
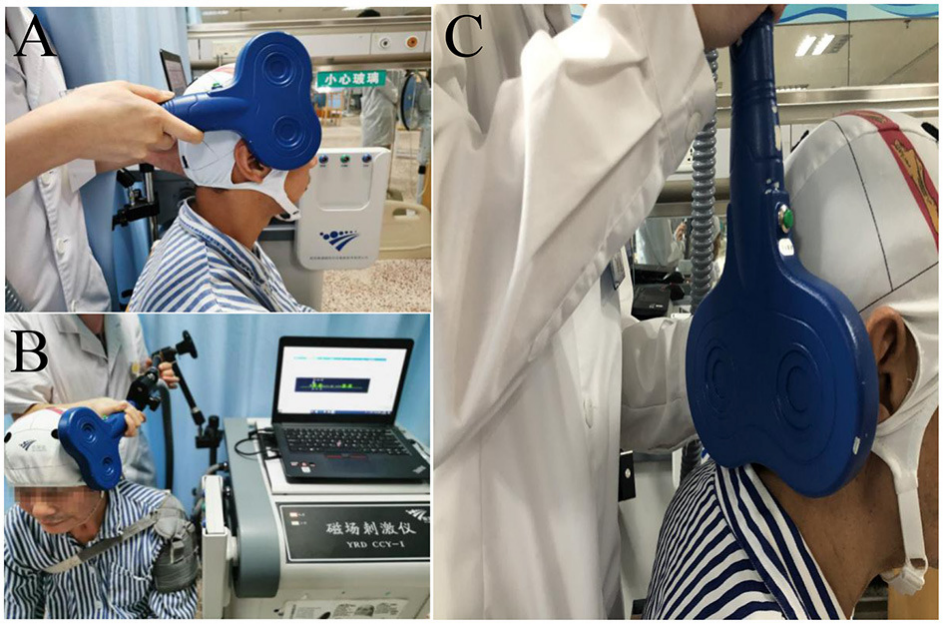
**(A)** For the affected hemispheric group, 5 Hz rTMS was applied at the affected mylohyoid cortical region. **(B)** For the unaffected hemispheric group, 5 Hz rTMS was applied at the unaffected mylohyoid cortical region. **(C)** For the cerebellum group, 5 Hz rTMS was applied at the mylohyoid cortical representation of the cerebellum (4.3 cm lateral and 2.4 cm below the inion).

This study was an observer-blind and random controlled trial. Patients were randomly divided into three groups by the random number table method. A sealed opaque envelope was opened at patient enrollment to determine whether the patient was to be assigned to the unaffected hemispheric, affected hemispheric or cerebellum group. These three groups of patients received 10 consecutive rTMS sessions for 2 weeks. For the affected hemispheric group and unaffected hemispheric group, 5 Hz rTMS was applied to the affected mylohyoid cortical region (Figure 2A) or to the unaffected mylohyoid cortical region (Figure 2B). For the cerebellum group, 5 Hz rTMS was applied to the mylohyoid cortical representation of the cerebellum (4.3 cm to lateral and 2.4 cm below the inion) (Figure 2C) (19). These three groups of patients received the same amount of traditional dysphagia treatment for 30 min daily after the intervention, such as thermal tactile stimulation, vocal cord exercises, Shaker exercises, Masako maneuvers, oropharyngeal muscle strengthening exercises, and tongue retraction exercises. These exercises were conducted 5 days a week for 10 days with the guidance of an experienced physical therapist. Meanwhile, patients treated with rTMS were compared with a population of 35 post-stroke patients (control group) suffering from dysphagia who did not receive rTMS. The 35 post-stroke patients only received traditional dysphagia treatment 5 days a week for 2 weeks.

### Determination of the Resting Motor Threshold (RMT)

#### Unaffected Hemispheric Group and Affected Hemispheric Group

Each patient in the affected hemispheric group and unaffected hemispheric group was seated in a quiet environment and relaxed state. Electromyography (EMG) data representing oral swallowing musculature from mylohyoid muscles were detected using the same methods as Hamdy et al. (23). MagPro CCY-I stimulator (purchased from YIRUIDE Company, Wuhan, China) was used for magnetic stimulations with a 9 cm outer diameter figure-eight coil.

Cortical excitability on both hemispheres separately of each patient, including the motor evoked potential (MEP) and resting motor threshold (rMT) were measured using single-pulse TMS. The coil was moved around in an area within 2–4 cm anteriorly and 4–6 cm laterally of the vertex of the cranium to locate the mylohyoid cortical region of the hemisphere to obtain the maximum MEP recording (23). The maximum MEP recording location was regarded as the “hot spot,” representing magnetic stimulation delivered to the area. Single-pulse TMS was then delivered to the “hot spot” with a 2% reduction in the output of the stimulator. The definition of the rMT is that in 10 consecutive trials of mylohyoid muscles, five trials can induce the minimum stimulus intensity of MEP > 50 μV. The “hot spot” was defined as an unaffected symmetrical hemisphere if MEPs were absent when the stroke-affected hemisphere was stimulated.

#### The Cerebellum Group

In previous studies, it has been identified that rTMS stimulation is effective regardless of which side of the cerebellum is stimulated (19, 24). For the cerebellum group, the coil was fixed at the mylohyoid cortical representation of the cerebellum (4.3 cm to lateral and 2.4 cm below the inion) (19). The rMT was determined by the rMT of the mylohyoid cortical area of the unaffected hemisphere.

### Repetitive Transcranial Magnetic Stimulation Application

The same parameters of stimulation were used for each intervention group. For each patient, 20 min rMT intensity with 5 Hz at 110% was applied at the “hot spot” area, which would last for 10 days with a total of 1,800 pulses per day. The protocols of rTMS applied in this study were strictly followed by the clinical safety guidelines for rTMS applications (25).

### Outcome Measurements

**Figure 3:**
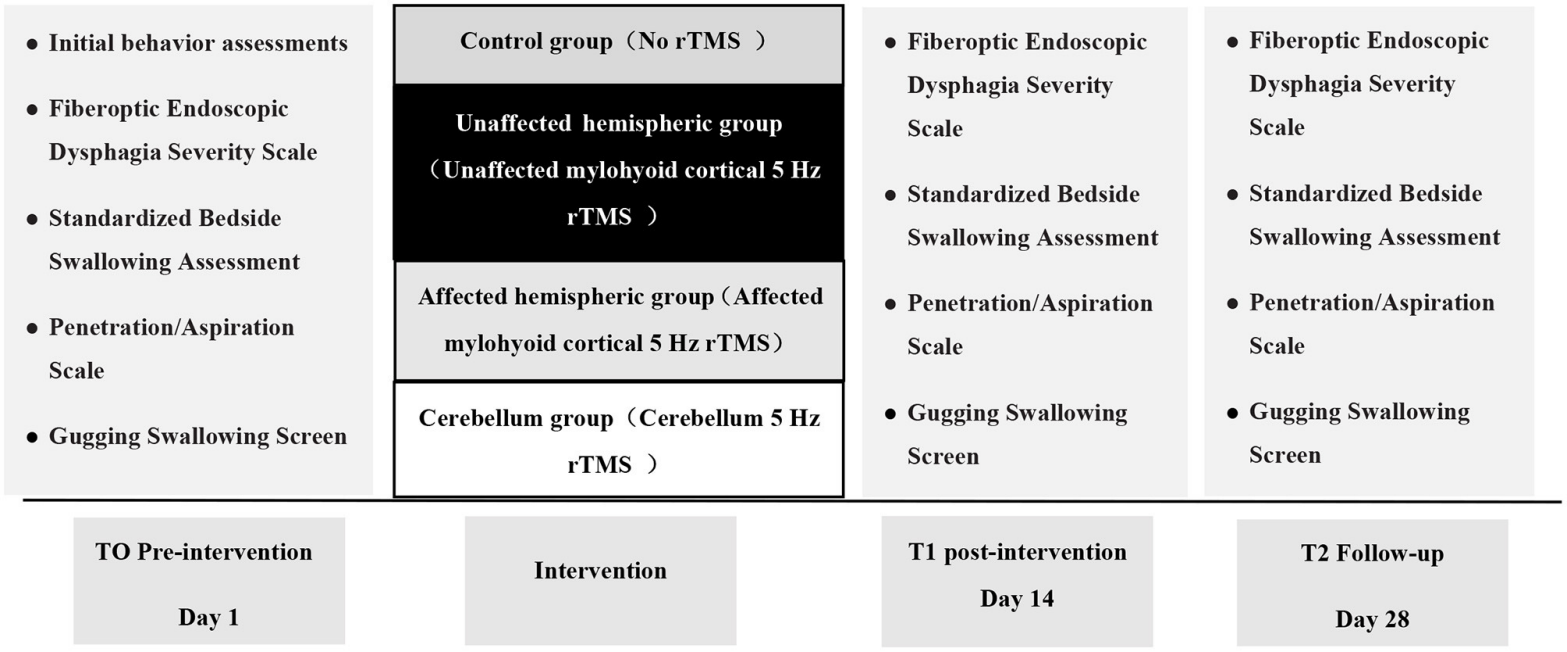
Experimental design.

All included participants were assessed at three different times: baseline (before the treatment), 2 weeks (after the treatment), and follow-up (2 weeks after the treatment) (see Figure 3). The primary outcome included the FEDSS scale; secondary outcomes involved assessments of the other dysphagia rating scales, such as the SSA scale, PAS scale, and GUSS scale.

### Fiberoptic Endoscopic Dysphagia Severity Scale (FEDSS)

All included patients required FEES. First, the secretion status of patients was measured, and then the patient received standard volumes of semiliquid diet, such as soft solid food, liquids, or puree. Stroke-related dysphagia was divided into a six-point FEDSS with 1 score for the best and 6 scores for the worst based on different consistencies of diet observed in the endoscopic examination and the risk of saliva penetration or aspiration (26).

### Standardized Bedside Swallowing Assessment (SSA)

The SSA consists of three parts. One section comprises eight indicators, including the responsiveness level, breathing, sound intensity, lip closure, control of trunk and head, voluntary cough and pharyngeal reflex. It is scored vary from 8 to 23 points. In the second section, the patients swallowed 5 mL water three times, and at the same time, salivary management and laryngeal movement were assessed. Repetitive swallowing, stridor, choking, and vocal quality were also evaluated, with a score range of 5–11 points. Once patients completed the first two parts of the assessment, they underwent the third part that entailed swallowing 60 mL water; this activity was scored from 5 to 12 points. The total SSA score varied from 18 to 46 points, and higher scores indicated worse swallowing function (27, 28).

### Penetration/Aspiration Scale (PAS)

Dysphagia severity was scored by an 8-point scale named the Penetration/Aspiration Scale (PAS). This scale was widely conducted for semiquantitative assessment of the degree of penetration and aspiration of endoscopic or radiological measurements, with higher scores indicating more severe impairment (29).

### Gugging Swallowing Screen (GUSS)

The GUSS is a validated reliable screening test for swallowing with a maximum score of 20. This tool consists of two parts: five indirect questions were used to measure the swallow function of the patient, and four direct questions were conducted to assess the physical condition of patients when ingesting liquid, semisolid and solid food. A higher score suggested a milder condition of dysphagia, but a lower score suggested a more serious dysphagia condition. Fourteen points were deemed passing scores for swallowing, and patients who scored <14 points were regarded as having a high likelihood of aspiration (30).

### Statistical Analysis

In this study, statistical analyses were conducted with SPSS 23.0 software (SPSS Inc., Chicago, IL, USA). Two-way analysis of variance (ANOVA) was used for continuous data among multigroup comparisons (normal distribution), and the chi-squared test was performed for categorical data. To assess the effect of the interaction between intervention and time, repeated measure analysis of variance (ANOVA) was used, in which time was used as a within-subject factor and intervention as a between-subject factor. *Post-hoc* analysis was performed using Bonferroni correction. A Greenhouse-Geisser correction was performed to correct the non-sphericity of the data. A *P* < 0.05 was considered significantly different.

## Results

One hundred forty-seven subjects were randomized into four groups. The average ages in the unaffected hemisphere group, the affected hemisphere group, the cerebellum group and the control group were 64.47 ± 13.95 years (28 males and 10 females), 64.67 ± 10.87 years (28 males and 8 females), 63.18 ± 9.92 years (20 males and 14 females), and 62.34 ± 11.54 years (18 males and 17 females), respectively. There were no significant differences between the groups at baseline in clinical and demographic characteristics, Basic Activities of Daily Living (BADL) score, Mini-Mental State Examination (MMSE) score, Eating Assessment Tool-10 (EAT-10) score, Nutrition Risk Screening-2002 (NRS2002) score, Water Swallow Test (WST) score, FEDSS score, PAS score, SSA score, or GUSS score (Table 1).

**Table 1:** The demographic and clinical characteristics of the included patients.

	**Unaffected *N* = 38**	**Affected *N* = 36**	**Cerebellum *N* = 34**	**Control *N* = 35**	***P***
Sex (F:M)	10: 28	8: 28	14: 20	17: 18	0.063
Age (years)	64.47 ± 13.95	64.67 ± 10.87	63.18 ± 9.92	62.34 ± 11.54	0.814
Type of stroke (Hemorrhage: Ischemia)	18: 20	12: 24	10: 24	14: 21	0.411
Affected hemisphere (Right: Left: infratentorial)	10: 20: 8	10: 14: 12	6: 12: 16	5: 15: 15	0.265
Duration of onset of stroke (days)	30 (15–60)	18 (14–60)	20 (14.25–30)	25 (15–30)	0.433
BADL	28.95 ± 21.91	26.94 ± 22.62	21.47 ± 23.08	23.71± 20.66	0.489
MMSE	13.84 ± 6.71	17.43 ± 8.35	15.02 ± 6.43	14.60 ± 7.57	0.182
EAT-10	17.70 ± 8.72	17.84 ± 10.09	18.84 ± 6.76	18.89± 8.64	0.890
NRS 2002	3 (2–4)	2.5 (2–4)	3.25 (2.75–3.44)	3 (2–4)	0.412
WST	4 (3–5)	4 (3–5)	4 (4–5)	4 (4–5)	0.089
FEDSS	3.68 ± 0.93	3.69 ± 1.19	4.06 ± 0.95	4.06 ± 0.76	0.168
PAS	5.47 ± 1.64	5.19 ± 1.79	5.91 ± 1.38	5.46 ± 1.54	0.311
SSA	27.79 ± 4.83	27.61 ± 4.99	27.56 ± 4.35	27.71 ± 3.50	0.996
GUSS	6.42 ± 5.52	5.72 ± 4.77	5.59 ± 4.77	5.60 ± 4.91	0.874

*Data are described as the mean ± SD or median (interquartile range). FEDSS, Fiberoptic Endoscopic Dysphagia Severity Scale; MMSE, Mini-Mental State Examination; PAS, Penetration/Aspiration Scale; WST, Water Swallow Test; SSA, Standardized Swallowing Assessment; BADL, Basic Activities of Daily Living; GUSS, Gugging Swallowing Screen*.

**Figure 4:**
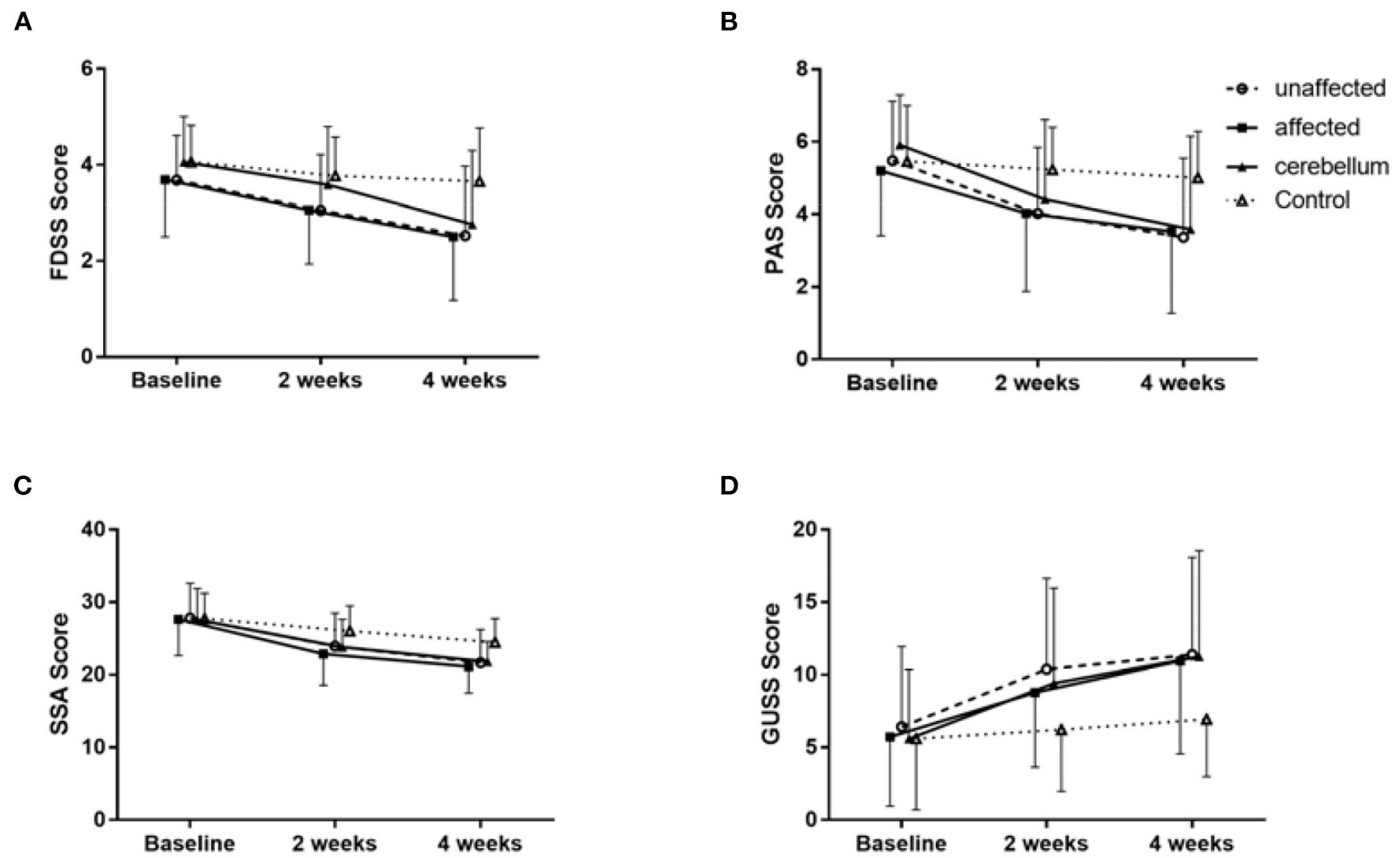
Changes in the mean rating scores of FEDSS **(A)**, PAS **(B)**, SSA **(C)**, and GUSS **(D)** at the three evaluation points in the four groups of patients. Data are described as the mean ± SD. Each group showed significant improvement separately.

Compared with baseline, the FEDSS and PAS scores of all groups improved at 4 weeks. The FEDSS scores were significantly different at 2 weeks (*P* = 0.008) and 4 weeks (*P* = 0.001). Similarly, there was a significant difference in PAS scores at 2 weeks (*P* = 0.024) and 4 weeks (*P* = 0.005) (Table 2). Figures 4A,B showed FEDSS and PAS scores at each time point in the four groups.

**Table 2:** Clinical rating scales (FEDSS, PAS, SSA, and GUSS) for the four groups at each time.

		**Unaffected**	**Affected**	**Cerebellum**	**Control**	***P*-value**
FEDSS						
Baseline		3.68 ± 0.93	3.69 ± 1.19	4.06 ± 0.95	4.06 ± 0.76	0.168
2 weeks		3.05 ± 1.16	3.06 ± 1.12	3.59 ± 1.21	3.77 ± 0.81	0.008
4 weeks		2.53 ± 1.45	2.50 ± 1.32	2.76 ± 1.54	3.66 ± 1.11	0.001
PAS						
Baseline		5.47 ± 1.64	5.19 ± 1.79	5.91 ± 1.38	5.46 ± 1.54	0.311
2 weeks		4.03 ± 1.82	4.03 ± 2.16	4.41 ± 2.20	5.23 ± 1.17	0.024
4 weeks		3.37 ± 2.17	3.53 ± 2.26	3.59 ± 2.56	5.00 ± 1.28	0.005
SSA						
Baseline		27.79 ± 4.83	27.61 ± 4.99	27.56 ± 4.35	27.71 ± 3.50	0.996
2 weeks		23.92 ± 4.57	22.86 ± 4.32	23.79 ± 3.83	26.03 ± 3.49	0.012
4 weeks		21.66 ± 4.58	21.11± 3.66	21.79 ± 2.78	24.46 ± 3.27	0.001
GUSS						
Baseline		6.42 ± 5.52	5.72 ± 4.77	5.59 ± 4.77	5.60 ± 4.91	0.874
2 weeks		10.37 ± 6.28	8.78 ± 5.14	9.41 ± 6.57	6.23 ± 4.26	0.017
4 weeks		11.37 ± 6.72	10.94 ± 6.38	11.24 ± 7.32	6.94 ± 3.95	0.008
	**Unaffected vs. affected (*****P*****-value)**	**Unaffected vs. cerebellum (*****P*****-value)**	**Affected vs. cerebellum (*****P*****-value)**	**Unaffected vs. control (*****P*****-value)**	**Affected vs. control (*****P*****-value)**	**Cerebellum vs. control (*****P*****-value)**
FEDSS						
Baseline	1.000	0.631	0.718	0.625	0.712	1.000
2 weeks	1.000	0.232	0.254	0.033	0.038	1.000
4 weeks	1.000	1.000	1.000	0.003	0.003	0.044
PAS						
Baseline	1.000	1.000	0.375	1.000	1.000	1.000
2 weeks	1.000	1.000	1.000	0.043	0.048	0.442
4 weeks	1.000	1.000	1.000	0.008	0.024	0.039
SSA						
Baseline	1.000	1.000	1.000	1.000	1.000	1.000
2 weeks	1.000	1.000	1.000	0.176	0.008	0.148
4 weeks	1.000	1.000	1.000	0.008	0.001	0.018
GUSS						
Baseline	1.000	1.000	1.000	1.000	1.000	1.000
2 weeks	1.000	1.000	1.000	0.013	0.354	0.123
4 weeks	1.000	1.000	1.000	0.017	0.046	0.029

*FEDSS, Fiberoptic Endoscopic Dysphagia Severity Scale; GUSS, Gugging Swallowing Screen. SSA, Standardized Swallowing Assessment; PAS, Penetration/Aspiration Scale*.

After 2 weeks of rTMS treatment, the improvement of dysphagia in the unaffected hemisphere group, the affected hemisphere group and the cerebellum group was significantly better than that in the control group. For the FEDSS, repeated measure analysis of variance showed a significant main effect of assessment time point (F = 86.106, df = 1.724, *P* < 0.001) and a significant time–group interaction (F = 3.889, df = 5.173, *P* = 0.002) (Table 2; Figure 4A).

The SSA and GUSS scores of all patients improved during the follow-up. There were significant differences in SSA scores at 2 weeks (*P* = 0.012) and 4 weeks (*P* = 0.001) (Table 2; Figure 4C). Similarly, at 2 weeks (*P* = 0.017) and 4 weeks (*P* = 0.008), the GUSS scores were significantly different. Repeated measure analysis of variance showed a significant main effect of the assessment time point (F = 87.728, df = 1.416, *P* < 0.001) and a significant interaction (time-group) for the GUSS (F = 5.122, df = 4.372, *P* < 0.001; Figure 4D).

Three participants (one unaffected and two affected) suffered transient headache. No participants developed seizures during or after therapy.

## Discussion

**Figure 5:**
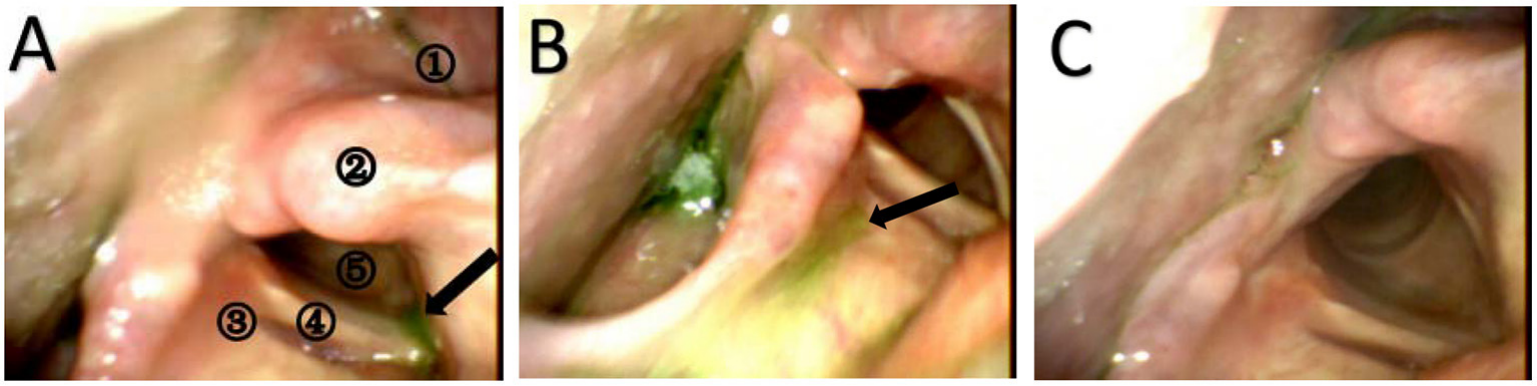
Still images from the FEES examination of a 66-year-old man with dysphagia at three different times. **(A)** FEES examination before the treatment (baseline). The black arrow represents aspirated puree in the subglottis. The patient does not try to cough and clear the material. Therefore, the FEDSS score is 5 points, and the PAS score is 8 points. A1 = pyriform sinus, A2 = arytenoid, A3 = laryngeal vestibule, A4 = vocal fold, A5 = subglottic. **(B)** FEES examination after the treatment (2 weeks). Puree is attached to the laryngeal vestibule, and the patient tries to cough but cannot clear it. The FEDSS score was 4 points, and the PAS score was 3 points. **(C)** FEES examination at the time of follow-up. Food is not inhaled into the laryngeal vestibule or subglottis. The FEDSS score and PAS score were both 1 point.

Our study compared the effects of dysphagia intervention based on the stimulation site: the affected mylohyoid cortical area, unaffected mylohyoid cortical area and cerebellum. This study revealed large effect sizes for swallow scores (FEDSS, PAS, SSA, and GUSS) after the end of intervention in the unaffected hemispheric group, the affected hemispheric group and the cerebellum group compared to the control group. These results suggest that rTMS stimulation of the affected hemisphere, unaffected hemisphere and cerebellum was useful in improving swallowing function in patients with dysphagia after stroke. Nevertheless, the effects among these sites were not significantly different. Figure 5 shows the changes in FEDSS and PAS scores in a patient treated with rTMS.

The mechanism of rTMS is not fully understood. Some previous studies (13, 31) were based on the hypothesis that the balance of activity between the hemispheres of the brain is perturbed after stroke, leading to impaired neurological function. Neurophysiologically, this interhemispheric imbalance is considered to be caused by altered transcallosal inhibition, with an abnormal increase in excitability in the contralesional hemisphere inhibiting the ipsilesional hemisphere. Therefore, in some previous studies (13, 31), rTMS has been used to restore the balance between the hemispheres of the brain to improve functional outcomes. In recent years, studies have confirmed that the projection of swallowing function in the human cerebral cortex is bilateral, with a dominant hemisphere that controls swallowing in patients with dysphagia (32, 33). High-frequency stimulation promotes cortical excitability, while low-frequency stimulation lowers excitability (34). rTMS can directly affect the cerebral cortex, effectively adjust the excitability of the cerebral cortex, reconstruct the central nervous system, form neural pathways, regulate swallowing centers, and improve swallowing function. Regarding the effects of cerebellar targeted rTMS, it is potentially interpreted that rTMS activates the cerebellar cortex, resulting in subsequent stimulation of dentate nuclei in each individual cerebellar hemisphere (24) because the functions of the cerebellum, which serves as a sensor and motor regulated organ, are predominantly suppressive (35). Hence, rTMS over the cerebellar cortices may lead to a decrease in inhibitory outflow and an increase in cortical activity. In this study, 5 Hz rTMS stimulation of the affected hemisphere, unaffected hemisphere and cerebellum may have facilitated swallowing function by improving cortical excitability of the mylohyoid cortex.

Previous studies have shown different outcomes in which various stimulation parameters of rTMS could improve the function of dysphagia in patients after stroke. For example, Park et al. (9) showed that high-frequency (5 Hz) rTMS application on the contralesional pharyngeal motor cortex was beneficial for poststroke dysphagic patients. Khedr et al. (11) proved that rTMS with 3 Hz high frequency at the lesional pharyngeal motor cortex resulted in significant improvement in dysphagia compared to a sham-stimulated group. These studies indicate that contralesional and lesional pharyngeal motor rTMS stimulation are both beneficial for reducing poststroke dysphagia. This is consistent with our research showing that rTMS stimulation at a high frequency in the unaffected hemisphere and affected hemisphere could significantly promote dysphagia recovery compared with the control group. The recovery of swallowing function may be related to changes in cortical excitability and neuroplasticity. Increases in cortical excitability by application of 5 Hz rTMS may increase stimulation to the motor neurons in the corticobulbar and corticospinal tracts, which enhances the synaptic innervations that project to the mylohyoid muscles, improves the movement of mylohyoid muscle, and promotes the recovery of swallowing function. Further neuroimaging tests or neurophysiologic evaluation are needed to delineate the underlying neuromechanism. Overall, our study and previous studies indicate that high-frequency rTMS stimulation of mylohyoid cortical tissue benefits poststroke dysphagia.

Recently, a growing number of studies have explored the possibility of rTMS on cerebellar tissue in the treatment of dysphagia. Some studies (19, 36) have shown that hemispheric cerebellar rTMS can increase cortical PMEP amplitudes. Vasant et al. (20) found that active cerebellar rTMS can increase PMEP amplitude, and their results indicated that cerebellar rTMS is a safe method that represents a potential treatment for poststroke dysphagia. Sasegbon et al. (24) demonstrated that high-frequency rTMS on the cerebellum could reverse the disruptive effects of a “virtual lesion.” These findings provide evidence for the development of cerebellar rTMS as a treatment for dysphagia after stroke. Our findings showed that rTMS stimulation at a high frequency in the cerebellum could significantly promote dysphagia recovery compared with the control group. However, one study (37) showed that, compared with unilateral stimulation, bilateral cerebellar rTMS has a greater promotion effect on corticobulbar motor pathways to the pharynx and may be a more effective clinical therapy. Another study (19) found that 10 Hz rTMS seems to be the best frequency to promote excitement of the pharyngeal motor cortex. At present, the optimal stimulation parameters of rTMS on cerebellar tissue are still uncertain. More clinical trials are needed in the future to further improve the technology.

Recent studies show that compared to unilateral stimulation, bilateral pharyngeal stimulation with 10 Hz rTMS stimulation on “hot spots” has more positive outcomes in both acute and chronic stroke patients (38, 39). However, these trials did not compare the effects of ipsilesional and contralesional rTMS. Furthermore, they did not compare the effects of cerebellar rTMS to cerebral hemispheric rTMS. To the best of our knowledge, our study was the first to directly compare the therapeutic impact of high-frequency rTMS applications on the unaffected hemisphere, affected hemisphere and cerebellum to evaluate the effects on swallowing function applications in stroke patients. Our findings show no difference, based on FEDSS, PAS, SSA, and GUSS outcomes, among the affected hemisphere, unaffected hemisphere and cerebellum. Similarly, there was no statistically significant difference between the groups in the subgroup analysis of a meta-analysis according to intervention site (ipsilesional vs. contralesional site stimulation) (14). However, another meta-analysis reported that contralesional stimulation is better than ipsilesional stimulation (15). The meta-analysis involved interventions that included non-invasive brain stimulation, either rTMS or tDCS. The pooled effect showed high heterogeneity concerning dysphagia evaluations, population, stroke etiology, clinical characteristics of stroke, and intervention time after stroke onset. Therefore, more rigorously designed original studies are necessary to identify the effects of different stimulation sites.

This study may possess the following limitations. First, the difference in swallowing function rehabilitation by stroke type was not analyzed. We were not able to perform cerebellar subgroup analysis according to affected, unaffected and cerebellar stroke lesions on account of the insufficient number of patients with infratentorial stroke lesions. Second, the effect of rTMS in our study was evaluated based on the clinical severity and fiberoptic endoscopic dysphagia severity scale and not on neurophysiologic evaluation, such as MEP amplitude and latency of rTMS. Finally, the effect of rTMS on brain plasticity was not evaluated by neuroimaging tests or neurophysiologic evaluation in our study. In the future, the combination of neuroimaging studies and neurophysiology would be beneficial in exploring the potential mechanism of rTMS in the recovery of dysphagia.

## Conclusions

The present study suggested that 5 Hz rTMS in the affected hemisphere, unaffected hemisphere and cerebellum for 10 days improves swallowing function in poststroke dysphagia patients. However, no difference among the affected hemisphere, unaffected hemisphere and cerebellum was observed. Therefore, regardless of whether the unaffected hemisphere or the affected hemisphere is stimulated, 5 Hz high-frequency rTMS on mylohyoid cortical tissue might have a positive effect on patients with poststroke dysphagia. In addition, cerebellar rTMS is a safe method that represents a potential treatment for poststroke dysphagia, and more clinical trials are needed to further improve this technique.

## Data Availability Statement

The raw data supporting the conclusions of this article will be made available by the authors, without undue reservation.

## Ethics Statement

The studies involving human participants were reviewed and approved by the Ethics Committee of Yue Bei People's Hospital. The patients/participants provided their written informed consent to participate in this study. Written informed consent was obtained from the individual(s) for the publication of any potentially identifiable images or data included in this article.

## Author Contributions

HL contributed to the conception of the study, supervised the clinical trial, and performed manuscript writing and editing. LZ, JW, and JR performed data analyses and manuscript writing and editing. PW and YZ contributed to the conception and design of the study. FL and YP performed data collection. All authors have agreed with the submitted version of the manuscript.

## Conflict of Interest

The authors declare that the research was conducted in the absence of any commercial or financial relationships that could be construed as a potential conflict of interest.
